# On the setting of environmental noise and the performance of population dynamical models

**DOI:** 10.1186/1472-6785-10-7

**Published:** 2010-03-12

**Authors:** Crispin M Mutshinda, Robert B O'Hara

**Affiliations:** 1 Department of Mathematics and Statistics, PO Box 68 (Gustaf Hällströmin katu 2b), FIN-00014 University of Helsinki, Finland; 2 Biodiversity and Climate Research Centre, Senckenberganlage 25, D-60325 Frankfurt am Main, Germany

## Abstract

**Background:**

Environmental noise is ubiquitous in population growth processes, with a well acknowledged potential to affect populations regardless of their sizes. It therefore deserves consideration in population dynamics modelling. The usual approach to incorporating noise into population dynamical models is to make some model parameter(s) (typically the growth rate, the carrying capacity, or both) stochastic and responsive to environment fluctuations. It is however still unclear whether including noise in one or/and another parameter makes a difference to the model performance. Here we investigated this issue with a focus on model fit and predictive accuracy. To do this, we developed three population dynamical models of the Ricker type with the noise included in the growth rate (Model 1), in the carrying capacity (Model 2), and in both (Model 3). We generated several population time series under each model, and used a Bayesian approach to fit the three models to the simulated data. We then compared the model performances in fitting to the data and in forecasting future observations.

**Results:**

When the mean intrinsic growth rate, *r*, in the data was low, the three models had roughly comparable performances, irrespective of the true model and the level of noise. As *r *increased, Models 1 performed best on data generated from it, and Model 3 tended to perform best on data generated from either Models 2 or Model 3. Model 2 was uniformly outcompeted by the other two models, regardless of the true model and the level of noise. The correlation between the deviance information criterion (DIC) and the mean square error (MSE) used respectively as measure of fit and predictive accuracy was broadly positive.

**Conclusion:**

Our results suggested that the way environmental noise is incorporated into a population dynamical model may profoundly affect its performance. Overall, we found that including noise in one or/and another parameter does not matter as long as the mean intrinsic growth rate, *r*, is low. As *r *increased, however, the three models performed differently. Models 1 and 3 broadly outperformed Model 2, the first having the advantage of being simple and more computationally tractable. A comforting result emerging from our analysis is the broad positive correlation between MSEs and DICs, suggesting that the latter may also be informative about the predictive performance of a model.

## Background

Population fluctuations typically result from the interplay between demographic stochasticity caused by chance variation in survival and reproduction events among individuals in a finite population, density-dependent feedbacks, and environmental noise or environmental forcing induced by temporal fluctuations in the environment experienced by individual organisms [1,2]. Whilst demographic stochasticity tends to average out with the population size and remains important only in small populations, environmental stochasticity affects populations regardless of their sizes [1,3-5]. It therefore deserves consideration in both descriptive and predictive settings. In the current phase of global climatic changes, evaluating the ecological consequences of environmental forcing has become a critical issue in ecology. However, there is still considerable uncertainty as to the most appropriate way of incorporating environmental noise into population dynamical models. The usual approach is to make model parameter(s), typically the growth rate [6,7] or the carrying capacity [8-10], stochastic and responsive to environmental perturbations. It has also been suggested [11] that, from a biological point of view, assuming variability in both parameters would be most realistic. However, not only does such an approach make it difficult to disentangle density-dependent feedbacks and stochastic noise in the data, it also introduces further computational issues.

Both theoretical and empirical studies have suggested that the way environmental noise is incorporated into population models may affect their behaviour. For example, Brännström & Sumpter [2] used the Ricker model [12] to show, through a simulation study, that including environmental noise in the growth rate or in the carrying capacity results in different population dynamical effects. Rockwood [[13], p. 56] simulated population time series under the Ricker model assuming stochasticity in the growth rate, in the carrying capacity, and in both. He found that populations with stochastic growth rate became stable around the carrying capacity. Populations with stochastic carrying capacity went through a series of crashes, and populations in which both the growth rate and the carrying capacity were allowed to vary also went through crashes and tended to go extinct more often, due presumably to larger total variance. However, Rockwood only considered a dynamical regime where the deterministic dynamics were stable. The model performance may be different for example, when the dynamics are cyclic or chaotic. The magnitude of environmental noise may also have far-reaching implications for the model behaviour.

Here we conduct a simulation study to investigate whether including noise in one or/and another parameter makes a difference to the model explanatory and predictive performances. We proceed by developing three population dynamics models of the Ricker type with the noise included in the growth rate (Model 1), in the carrying capacity (Model 2), and in both (Model 3). We simulate several population time series under each model with different magnitudes of noise ranging from low to high, and use a Bayesian approach [14] to fit the three models to the simulated data using Markov chain Monte Carlo (MCMC) methods [15]. We then utilize the deviance information criterion (DIC) [16] and the mean square error (MSE) to evaluate the performances of the three models in regard to their fit to the data and their forecast accuracy, respectively.

## Results

**Figure 1:**
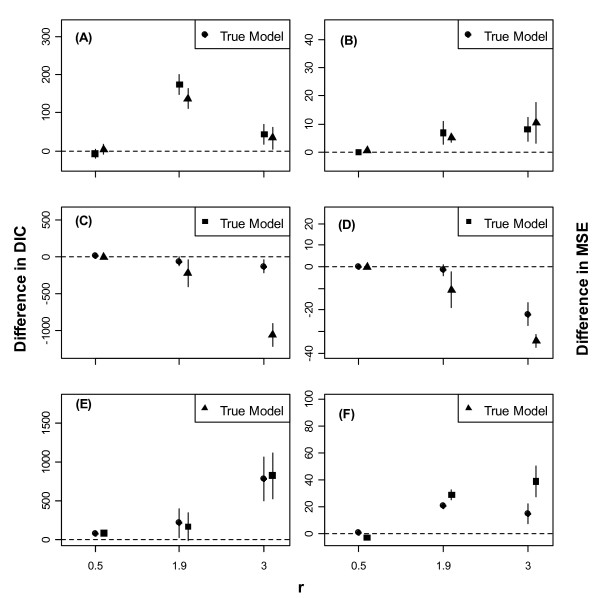
**Error-bars (mean ± 1SD) of differences in DIC between a model and the true (i.e., the data generating) model for low level of environmental noise in the data**. Error-bars (mean ± 1SD) of differences in DIC (1A, 1C & 1E) and in MSE (1B, 1D & 1F) between a model and the true (i.e., the data generating) model for low level of noise in the data. The filled circle represents Model 1, the filled square Model 2, and the filled triangle Model 3. The dashed horizontal line corresponds to an agreement between the true and the contending model.

**Figure 2:**
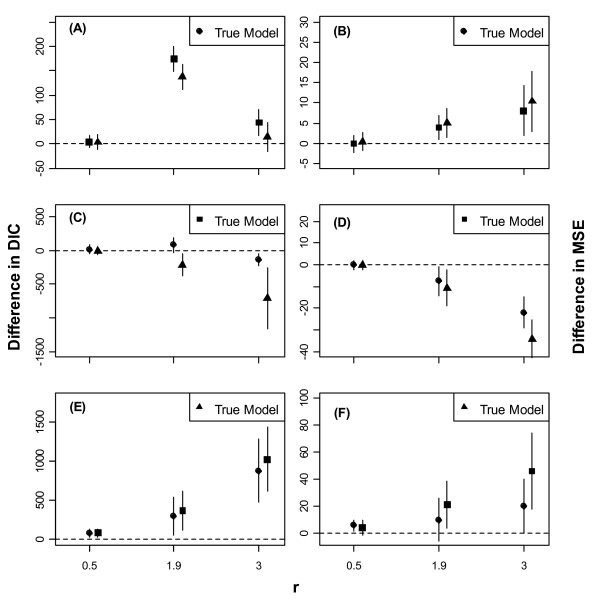
**Error-bars (mean ± 1SD) of differences in DIC between a model and the true (i.e., the data generating) model for moderate level of environmental noise in the data**. Error-bars (mean ± 1SD) of differences in DIC (2A, 2C & 2E) and in MSE (2B, 2D & 2F) between a model and the true (i.e., the data generating) model for moderate level of noise in the data. Models are identified by the same symbols are as in Fig 1. The dashed horizontal line corresponds to an agreement between the true and the contending model.

**Figure 3:**
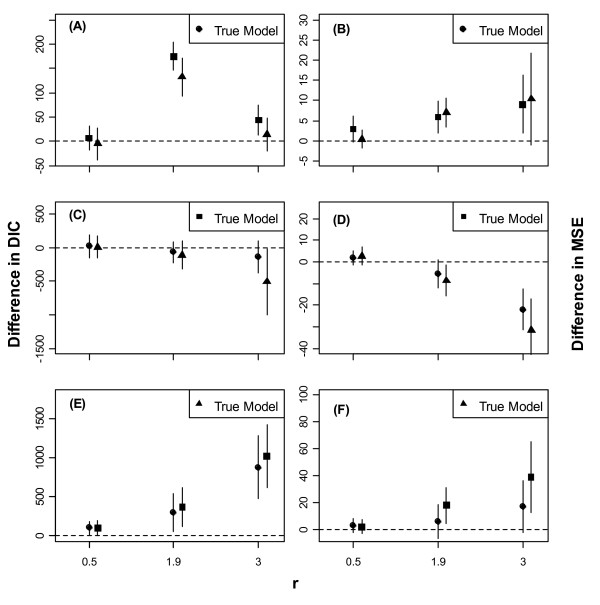
**Error-bars (mean ± 1SD) of differences in DIC between a model and the true (i.e., the data generating) model for high level of environmental noise in the data**. Error-bars (mean ± 1SD) of differences in DIC (3A, 3C & 3E) and in MSE (3B, 3D &3F) between a model and the true (i.e., the data generating) model for high level of noise in the data. Models are identified by the same symbols are as in Fig 1. The dashed horizontal line corresponds to an agreement between the true and the contending model.

Figs. 1, 2 and 3 show error-bars (mean ± SD) for differences in DICs and MSEs between a model and the true (i.e., the data generating) model for low, moderate, and high noise in the data, respectively.

When the mean intrinsic growth rate in the data, *r*, was low, the three models had approximately comparable performances irrespective of the true model and the level of noise (Figs. 1, 2 and 3). As *r *increased, however, Models 1 and 3 outperformed Model 2, even when Model 2 was the true model, regardless of the level of noise.

**Figure 4:**
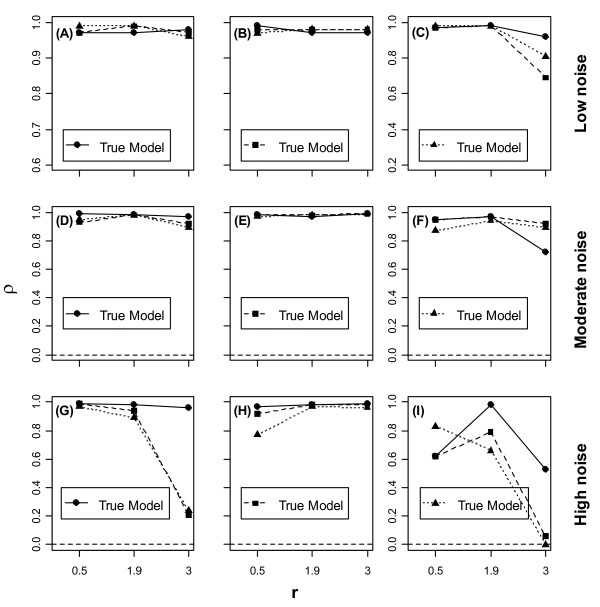
**Correlations between deviance information criteria and mean square errors for different parameter settings**. Correlations between deviance information criteria (DICs) and mean square errors (MSEs) for different models under different parameter settings and different magnitudes of environmental noise; models are identified by the same symbols are as in Fig 1.

Fig. 4 shows correlations between DICs and MSEs across the three models for data generated under different models, different dynamical regimes, and different magnitudes of noise in the data ranging from low to high.

The correlations were broadly positive under low and moderate noise (Figs. 4A-F). For high noise, however, the correlation tended to fade out under Model 2 and Model 3 (Figs. 4G &4I) as *r *exceeded 2, except in the case where the underlying process included noise in the carrying capacity only (Fig 4H).

## Discussion

Evaluating the ecological consequences of environmental forcing is a critical contemporary issue in ecology. However, the difficulties involved in effectively modelling ecological processes in random environments are widely recognized [17-19]. While environmental noise is typically incorporated into population dynamical models by making one or more model parameters stochastic and responsive to environmental disturbances, it is still unclear whether including noise in one or another parameter makes a difference to the model performance. In this paper we used the stochastic Ricker model to investigate this issue with a focus on model fit and predictive accuracy.

Although including noise in both parameters may be biologically more pragmatic [11], such an approach makes it difficult to differentiate between density-dependent compensation and stochastic noise in the data, and increases the computational burden. Considering the cases of stochastic growth rate and stochastic carrying capacity separately helps reduce the computational burden and allows to tease apart density-dependent feedbacks and environmental forcing [11], thereby allowing for their effects to be more accurately assessed.

The types of underlying dynamics may also impinge on the performance of the fitted models. It is obvious that if we only simulated data assuming low *r *values, we would have reached the conclusion that, as to the model fit and predictive performance, it does not matter whether the noise is included in anyone parameter or in both. However, this turned out to be the case for this particular setting only. It is worth emphasizing that whilst the Ricker model accommodates density-dependence through the use of a parameter, *K*, representing the carrying capacity, the strength of density-dependent effects is regulated by the intrinsic growth rate, *r*, with lower values of *r *corresponding to weak density compensation [20,21]. Gabriel & Burger [22] pointed out that if the population growth rate *r *exceeds an optimal value, then population sizes can overshoot the carrying capacity, thereby increasing the population extinction risk.

Our results suggest that, with regard to model fit and predictive performance, it does not matter whether noise is included in the growth rate, in the carrying capacity or in both, provided that the mean intrinsic growth rate, *r*, is low. As *r *gets larger, however, the model with stochastic carrying capacity (Model 2) tends to perform worse. The model including stochasticity in the growth rate only (Model 1) may be preferable since it is parsimonious and more computationally tractable and yet, has comparable performance to Model 3 in which both parameters are made stochastic. On a logarithmic scale, Model 1 boils down to a nonlinear first-order autoregressive model for which many standard estimation tools are available [23,24]. Model 3 may be interesting if fluctuations in the carrying capacity can be constrained e.g., by imposing a suitable (informative) prior distribution on it. In line with [13], we found that even small noises in the carrying capacity tend to be magnified through density compensation when *r *is high, so that Model 2 can undergo large fluctuations. We also found that high noise decreases the model predictive accuracy as exemplified by the wider prediction intervals in Fig. 3, and tends to break the correlation between the DIC and MSE used here as measures of model fit and predictive accuracy, respectively (Fig. 4G &4I). One way of reducing noise and improve the model predictive accuracy is to incorporate suitable environmental covariates into the model.

From a statistical model fitting perspective, our results are comforting in two respects. First, they are consistent across simulations, i.e. when one model fits a simulated scenario better, it does so consistently, meaning that our conclusions can be generalized to similar data, something which may not always be so [19]. Second, the correlation between the measures of model fit (the DIC) and predictive accuracy (the MSE) was generally high (Fig. 4). Again, there is no guarantee that this will happen [25]. This suggests that we can have some confidence when comparing models using the DIC that this will be informative about their predictive abilities, the exception being when the dynamics are chaotic or highly noisy. This is because chaotic dynamics are inherently unpredictable. On the other hand, excessive noise can push a system from stability into chaos [26,27]. It is worth emphasizing that chaos is a characteristic not of an empirical system, but of a model we might have for it [26]. However, it has been suggested [28,29] that chaotic dynamics are atypical in nature. Although models may allow various dynamical regimes, the environment often constrains the dynamics that effectively occur.

We used simulated data in this work so that we would know what the true model was. For real data this is obviously not the case. But it does not matter since each model was fitted to data generated under different scenarios, the true model being used merely for comparison. Moreover, our analyses have shown that there is no guarantee that a model would perform best on data generated from it. In practice, model formulation should be guided by the knowledge of the system, the model purpose, and the nature of the data at hand. Model validation, e.g. through posterior predictive checking [14], should be a necessary step before a model can be used for further analyses.

## Conclusions

The development of flexible stochastic population dynamical models that can adjust to different data sets is an issue of practical relevance to ecology and conservation biology. However, the challenges involved in modelling ecological processes in random environments are widely documented [17-19]. In particular, it is unclear whether including noise in one or another parameter makes a difference to the performance of population dynamical models. In this paper we used the stochastic Ricker model to investigate this issue with a focus on the model performance in fitting to the data and in predicting new observations.

Our results suggested that the way noise is incorporated into a population dynamics model may greatly influence its performance. Overall, we found that including noise in one or/and another parameter does not affect the model performance as long as the mean intrinsic growth rate, *r*, is low. As *r *increased, however, different settings of environmental noise resulted in different model performances, meaning that in such a case, it becomes important to select the best model. The model including noise in the growth rate only is to favour since it performs as well as the model with stochasticity in both the growth rate and the carrying capacity, but has the advantage of being simple and more computationally tractable.

Our findings are clearly of relevance to conservation biology. Since the *modus operandi *of the processes underlying population fluctuations is unknown in practice, it is crucial to find flexible models that can easily adjust to different data sets. In this paper we provided useful guidelines for doing this. Our results highlight the necessity for cautious model selection when attempting to predict population dynamics e.g., in connection with conservational and management actions.

A comforting result emerging from our analysis is the broad positive correlation between the MSE and the DIC, suggesting that the latter may be informative for model selection in terms of predictive accuracy, unless the dynamics are chaotic or highly noisy.

## Methods

### Bayesian inference and model selection

Bayesian inference [14] is an approach to statistics in which all forms of uncertainty are expressed in terms of probability. A Bayesian analysis starts with the formulation of a probability model *p*(*y*|*θ*) describing the distribution of the data, *y*, conditionally of the (often vector-valued) unknown parameter *θ*. A prior distribution, *p*(*θ*), is then required to represent the state of knowledge about plausible values of *θ*, before seeing the data. After observing some data, the likelihood function, *p*(*y*|*θ*), is used to update the prior distribution into a posterior distribution, *p*(*θ*|*y*), according to Bayes' formula:

The posterior distribution is the tool for Bayesian inference about all unknown quantities including model parameters (estimation), and as yet unobserved data (prediction).

The posterior predictive distribution, , for a future observation, , given the data, *y*, is defined as . It is obvious that  integrates the likelihood, , of the future observation over the uncertainty about the model parameters encoded in the posterior distribution *p*(*θ*|*y*).

Choosing amongst alternative models or scientific hypotheses is a fundamental problem faced by researchers in any scientific discipline. Model selection can be viewed as a wide-scale testing problem where models rather than parameters are of interest [30]. The most prominent Bayesian model selection techniques include Bayes factors [31-33], decision theoretic criteria [16] and cross-validation [34]. We do not use Bayes factors here because they can be difficult to compute in practice, and are numerically unstable when proper, but diffuse priors are used [35].

From a decision theoretic perspective, the model selection problem can be cast in terms of minimizing a loss function appropriate to the decision problem at hand. A general loss function based the likelihood function is the deviance defined as *D*(*y*, *θ*) = -2log(*L*(*y*|*θ*)), where *L*(*y*|*θ*) denotes the likelihood function and log(.) denotes the natural logarithm. *D*(*y*, *θ*) is minimized as the corresponding utility function, the (log) likelihood, is maximized. Consequently, model selection can proceed by minimizing the deviance, , where  is the maximum likelihood estimate (MLE) of *θ*.

However, the likelihood of a dataset increases with the number of fitted parameters so that more complex models will often be selected. To overcome this bias towards higher dimensional models, penalized likelihood measures have been proposed. In the classical setting, the most popular penalized likelihood criteria are the Akaike information criterion (AIC) [36], and the Bayesian information criterion (BIC) or Schwarz criterion [37], where *k *is the number of free parameters, and *n *is the sample size. In the Bayesian framework, the deviance information criterion (DIC) introduced by [16] with an approximate decision theoretic justification is widely used, and has been utilized in this study.

The DIC is defined as , where  i.e., the posterior mean of the deviance: , and , where  is the deviance evaluated at the posterior mean of the model parameters: .  is interpreted as a measure of fit, whereas the so-called effective number of parameters, *P*_*D*_, acts as a penalty for model complexity. The analogy between the AIC and the DIC is apparent when the latter is written in the form .

The DIC is easily calculated from MCMC samples. One simply computes  as the average of *D*(*θ*|*y*) over the posterior samples of *θ*, and  as the value of the deviance at the average of the posterior samples of *θ*. The DIC follows directly from these approximations.

The hallmark of all good models or scientific theories is good prediction. When models are designed for prediction, model selection should be based on forecast accuracy. An important approach for selecting models with best predictive accuracy is cross-validation [34], where part of the data are used to calibrate the model whereas other subsets of the data are used to evaluate the model's predictive accuracy through an appropriate discrepancy measure. We can illustrate this for a time series data  by fitting the model to the first *n *data points and then predicting for the next *s *= *N *- *n *points in the series. For models that are built on a first-order Markov structure, meaning that *p*(*y*_*t*_|*y*_1_,...,*y*_*t*-1_, *θ*) = *p*(*y*_*t*_|*y*_*t*-1_, *θ*), as is the case for us here, one single-step prediction is enough. Different statistics can be used to evaluate the discrepancy between the data and the model predictions. Here we utilize the mean squared error (MSE) i.e., the average of the squared differences between the model predictions and the actual values, as measure of predictive accuracy.

### Simulation study

Let *y*_*t *_denote the population size/density at time *t *(*t *= 1,...,*T*). We assume that the population dynamics evolve according to a Ricker kernel [12], the deterministic form of which is given by *y*_*t *_= *y*_*t*-1 _exp{*r*(1-*y*_*t*-1_/*K*)}, where, *r *is the intrinsic growth rate, and *K *is the carrying capacity of the environment. The model can be written as *y*_*t *_= *λ*_*t*-1 _(*y*_*t*-1_), where *λ*_*t*-1 _= exp{*r*(1 - *y*_*t*-1_/*K*)} is the (multiplicative) population growth rate from time *t *- 1 to time *t*.

The dynamic behavior of the deterministic Ricker model is well-known: the model is stable for 0 <*r *< 2, and as *r *increases within this range, the model moves from smooth approach to equilibrium through an oscillatory approach. When *r *> 2, the system undergoes sustained doubling cycles to finally reach chaos at *r *≅ 3 [38].

Our interest here is on the behaviour of the stochastic Ricker model as environmental noise is included in the growth rate or/and in the carrying capacity. We develop three models differing by which parameters are used to accommodate environmental noise. Model 1: *y*_*t *_= exp{*r*(1 - *y*_*t*-1_/*K*) + *ε*_*t*_},  includes noise as log-normal multiplicative disturbances to the growth rate. That is, , which can be written as *y*_*t *_= *y*_*t*-1 _exp{(*r *+ *ε*_*t*_) - *r y*_*t*-1_/*K*}. Model 2: *y*_*t *_= exp{*r*(1 - *y*_*t*-1_/*K*_*t*_)}, includes noise in the carrying capacity, where *K*_*t *_= exp(*k *+ *η*_*t*_) and . In Model 3: *y*_*t *_= exp{*r*(1 - *y*_*t*-1_/*K*_*t*_) + *ε*_*t*_}, both parameters are made stochastic.

We generated several population time series under each model, with values of *r *set to 0.5, 1.9 and 3, and without loss of generality, the expected value of the mean carrying capacity, *K*, was set to Euler's number *e *(i.e., *k *= log(*K*) = 1). We generated time series of length 100 with the parameters *σ*_*r *_and *σ*_*k *_tuned to achieve the desired magnitude of noise ranging from low (*σ*_*r *_= 0.0625, *σ*_*k *_= 0.125), to moderate (*σ*_*r *_= 0.125, *σ*_*k *_= 0.25), to high (*σ*_*r *_= 0.25, *σ*_*k *_= 0.5).

In the model fitting process, the models were set up with a normal likelihood  truncated to positive values, where *μ*_*t *_is defined as *μ*_*t *_= *y*_*t*-1 _exp{*r*(1 - *y*_*t*-1_/*K*) + *ε*_*t*_} under Model 1, *μ*_*t *_= *y*_*t*-1 _exp{*r*(1 - *y*_*t*-1_/*K*_*t*_)} under Model 2, *μ*_*t *_= *y*_*t*-1 _exp{*r*(1 - *y*_*t*-1_/*K*_*t*_) + *ε*_*t*_} under Model 3, and  is intended to capture the unexplained variation.

The models were fitted to the data with a Bayesian approach using the following priors:  i.e., only positive values of *r *were allowed; *K*_*t *_~ *InvGa*(1, 1);  ~ *InvGa*(1, 1);  ~ *InvGa*(1, 1), where *InvGa*(*u*, *v*) denotes the inverse gamma distribution with parameters *u *and *v*. We used MCMC methods through the Bayesian freeware OpenBUGS [39] to numerically simulate samples from the joint posterior of the model parameters.

In all cases, 4000 burn-in iterations of 3 Markov chains starting from dispersed initial values in the parameter space were followed by a further 16000 iterations thinned to each 20^th ^observation (i.e., we saved the values at every 20^th ^iteration to reduce autocorrelation between the samples). The convergence of the MCMC was assessed visually though examining the mixing of the three chains and the behaviour of sample autocorrelation plots.

Simulations were run for 1000 replications and for each replication, we monitored the posterior mean of the DIC and the MSE averaged over the observational time. These yielded full distributions of these measures which were then used for assessing the model performance in fitting to the data and in forecasting future data.

## Authors' contributions

CMM designed the study, drafted the manuscript, and carried out the analyses. RBO supervised the research and revised the manuscript. All authors have approved the final manuscript.
